# Dialysate White Blood Cell Change after Initial Antibiotic Treatment Represented the Patterns of Response in Peritoneal Dialysis-Related Peritonitis

**DOI:** 10.1155/2016/6217135

**Published:** 2016-08-30

**Authors:** Pichaya Tantiyavarong, Opas Traitanon, Piyatida Chuengsaman, Jayanton Patumanond, Adis Tasanarong

**Affiliations:** ^1^Division of Clinical Epidemiology, Faculty of Medicine, Thammasat University, Rangsit Campus, Pathum Thani, Thailand; ^2^Division of Nephrology, Department of Medicine, Thammasat University Hospital, Pathum Thani, Thailand; ^3^Banphaeo Hospital, Prommitr Branch, Bangkok, Thailand

## Abstract

*Background*. Patients with peritoneal dialysis-related peritonitis usually have different responses to initial antibiotic treatment. This study aimed to explore the patterns of response by using the changes of dialysate white blood cell count on the first five days of the initial antibiotic treatment.* Materials and Methods*. A retrospective cohort study was conducted. All peritoneal dialysis-related peritonitis episodes from January 2014 to December 2015 were reviewed. We categorized the patterns of antibiotic response into 3 groups: early response, delayed response, and failure group. The changes of dialysate white blood cell count for each pattern were determined by multilevel regression analysis.* Results*. There were 644 episodes in 455 patients: 378 (58.7%) of early response, 122 (18.9%) of delayed response, and 144 (22.3%) of failure episodes. The patterns of early, delayed, and failure groups were represented by the average rate reduction per day of dialysate WBC of 68.4%, 34.0%, and 14.2%, respectively (*p* value < 0.001 for all comparisons).* Conclusion*. Three patterns, which were categorized by types of responses, have variable rates of WBC declining. Clinicians should focus on the delayed response and failure patterns in order to make a decision whether to continue medical therapies or to aggressively remove the peritoneal catheter.

## 1. Introduction

Peritoneal dialysis-related peritonitis is a common complication in end stage renal disease patients treated with continuous ambulatory peritoneal dialysis (CAPD) [1]. The nationwide prevalence was one episode every 17.1 to 25.8 months [2–5]. Bacteria are the most causative organism; thus initial antibiotics should be started immediately after the diagnosis is made [6]. Delayed treatment may lead to undesirable events such as prolonged hospitalization, higher rate of treatment failure, long-term peritoneal membrane dysfunction, and increased mortality.

Physicians usually use clinical parameters such as abdominal pain and dialysate white blood cells (WBC) to monitor treatment response. Numerous studies demonstrated that if dialysate WBC are more than 100 cells/mm^3^ at day 5 after treatment, the chance of treatment failure is high [7–9]. Consequently, the International Society of Peritoneal Dialysis (ISPD) defined the term “refractory peritonitis” as failure of dialysate WBC clearance after five days of antibiotic treatment and recommended removing the peritoneal catheter and stopping dialysis therapy in order to prevent morbidity and mortality associated with treatment failure [10, 11]. However, in some places where the hemodialysis resource is limited or the dialysis catheter is not readily removable, clinicians might choose to continue antibiotic treatment in selected patients who may have a delayed response to the treatment even though the target of dialysate WBC after the fifth day was not reached. Moreover, some of these patients were later recovered from peritonitis even without catheter removal. In our practice, we usually observe that dialysate WBC reduction in each patient is quite different; some have rapidly declined WBC in a few days, but some have a slow pattern of WBC reduction or increasing WBC. We then hypothesized that the pattern of response to antibiotic treatment varies individually. Therefore we designed this study to explore the pattern of response to initial antibiotic treatment by categorizing patients into early response, delayed response, and failure groups according to the rate of dialysate WBC change and we also explored the factors associated with treatment response.

## 2. Materials and Methods

### 2.1. Study Design and Setting

This was a retrospective cohort study which was jointly conducted at Banphaeo Hospital and Thammasat University Hospital, Thailand. Consecutive episodes of CAPD peritonitis in adult patients between January 2014 and December 2015 were included. The study was approved by the human research ethics committee of Thammasat University (Faculty of Medicine).

### 2.2. Participants

The patients included in this study were over 18 years old with a diagnosis of peritoneal dialysis-related peritonitis, defined by at least 2 out of 3 following criteria: (1) abdominal pain or cloudy peritoneal fluid, (2) amount of dialysate WBC greater than 100 cells/mm^3^, and (3) the organism being identified by Gram stain or microbiological culture [6]. Episodes were excluded if medical records were not found or held incomplete records of dialysate WBC at the time of peritonitis diagnosis or contained less than three time records of dialysate WBC in the first five days after treatment.

Standard care was applied to all peritoneal dialysis patients. A Tenckhoff catheter was placed on the abdominal wall and the dialysate was a lactated-buffered glucose solution in a twin-bag connecting system (Baxter Healthcare or Fresenius Medical Care). Training programs were introduced when CAPD was starting. All patients ran 3–5 cycles of CAPD. Since patients' caregivers or the patients themselves had suspected an incident of peritonitis, they would contact a nurse by phone and come to the hospital as soon as possible. After assessment by CAPD nurses and doctors, dialysate would be collected and sent to a laboratory for WBC counts (5 mL in EDTA tube) and bacterial culture (10 mL in two blood culture bottles and 5 mL centrifuged sediment for solid media). Antibiotics and other treatments were prescribed following the current ISPD guideline and the duration of treatment was mostly between 14 and 21 days depending on the clinician's judgments. Clinical symptoms and dialysate WBC counts were routinely monitored once a day or every other day to assess the treatment response. Specific antibiotics were prescribed according to the culture result. If there was no clinical improvement or dialysate WBC was persistently high, the clinician would make a decision whether to use a salvage antibiotic regimen, such as carbapenem and vancomycin, or to remove the Tenckhoff catheter.

We categorized outcomes of treatment into 3 groups: early response, delayed response, and failure. Early response was defined by clinical improvement with dialysate WBC counts at less than 100 cells/mm^3^ within 5 days of antibiotic treatment. Delayed response was determined if the dialysate WBC gradually decreased but still persisted more than 100 cells/mm^3^ after 5 days of antibiotic treatment with success at the end of treatment. The failure group was patients who were not cured by antibiotics and changed to hemodialysis either temporarily or permanently. Patients who died due to peritonitis would be classified into failure group as well.

### 2.3. Patterns of Response Assessment

The primary outcome was the WBC changing patterns in five days after administration of antibiotics for all groups. Dialysate WBC were counted and differentiated by using an automatic machine. Data recorded included age, gender, diabetes status, HIV status, primary kidney disease, peritoneal dialysis vintage, episodes of peritonitis, body temperature, types of empirical antibiotics, microbiology, and outcomes of treatment.

### 2.4. Statistical Analysis

The characteristics of the early response, delayed response, and failure groups were profiled. Descriptive statistics were used depending on the types of variables. Continuous data were expressed by mean and standard deviation if their distributions were normal. Median and range were applied for skewed data. Continuous variables were compared by one-way analysis of variance or the Kruskal-Wallis *H* test as appropriate. Exact probability test and Chi-square test were used for categorical variables comparison. Furthermore, subgroups by types of organism were shown.

Serial measurements of dialysate WBC were plotted graphically for each group. Due to the right skewed distribution of peritoneal WBC in each day, we used natural logarithm transformation for normalizing this particular data. The linear patterns of WBC in logarithm scale were found over the time of treatment. To combat the disproportionate effect of individual patients with repeated peritonitis, we used the multilevel regression for numerical data to explore the slope of WBC change. Multiple comparison with Bonferroni adjustment for *p* value was used to demonstrate the statistical difference of each pattern. The constant rate of WBC reduction per day was achieved by applying the exponential function of the Beta coefficient in the model. Finally, the ratio was internally validated using a bootstrapping procedure with 1,000 random samples with replacement.

All statistical tests were two-sided. We considered *p* value of less than 0.05 to point out the level of significance. All statistical analyses and graphics were performed with Stata software version 14.0 (StataCorp).

## 3. Results

### 3.1. Characteristics

**Figure 1:**
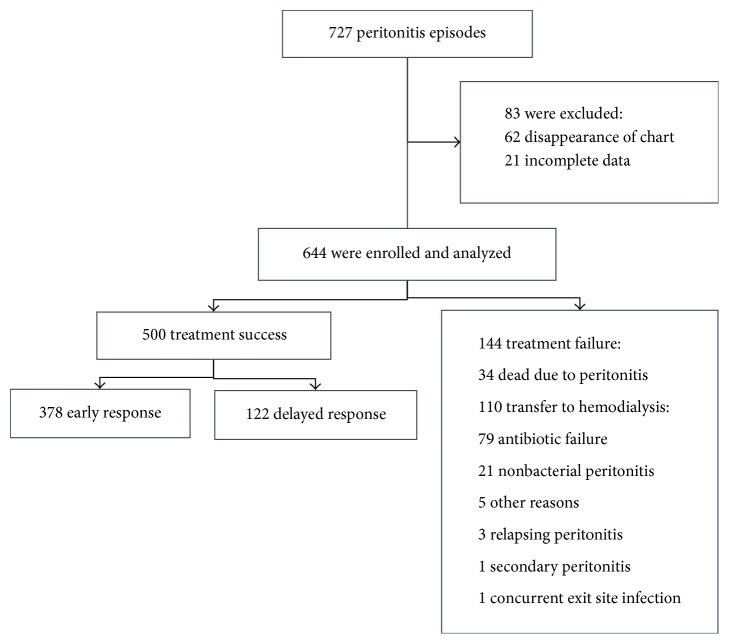
Study flow.

A total of 727 episodes of peritonitis were recorded between January 2014 and December 2015. Six hundred and forty-four episodes (from 455 patients) were included for analysis: 378 of early response (58.7%), 122 of delayed response (18.9%), and 144 of failure groups (22.3%). Thirty-four patients died due to peritonitis (mortality rate 5.3%) and the major causes of peritoneal dialysis termination were antibiotic failure (79 episodes) and nonbacterial peritonitis (21 episodes) (Figure 1).

Clinical characteristics were categorized by whether episodes of peritonitis had early response, delayed response, or treatment failure (Table 1). Two-thirds of our patients had diabetes. The failure group showed a higher median duration of peritoneal dialysis (19.8 months) and a lower percentage of the first episode of peritonitis (49.3%) but no statistical significance when compared with other groups. The usual empirical antibiotic regimen in our centers was intraperitoneal cefazolin (88.7%) and ceftazidime (89.9%). The rate of culture negative peritonitis which was about 30% in this cohort and types of organism differ significantly among the 3 groups (*p* value < 0.001). Gram-negative organism was found less in the early response group when compared with the delayed response group (24% versus 36.9%). Moreover, mixed organism and nonbacterial peritonitis appeared more frequently in the failure group. Empirical antibiotic success, defined as successful treatment without changing to any salvage antibiotic regimens, was found in all early response groups and 48.7 percent in delayed response groups in culture negative cases.

### 3.2. Types of Organism

Subgroup analysis was done according to the types of organism (Table 2). We found 75.6% of early response pattern in 213 episodes of single Gram-positive infection. This result suggested that, in general, if a Gram-positive organism was the causative pathogen, the response to initial antibiotic was usually good except for* Staphylococcus aureus* or methicillin-resistant* Staphylococcus aureus* (MRSA) infection (38.1% and 14.3%, resp.). In cases of single Gram-negative infection, the early response pattern was found only 52% in average. Delayed response pattern was accounted for one-fourth of single Gram-negative infection. Extended-Spectrum Beta-Lactamase- (ESBL-) producing* Escherichia coli* had the worst response to antibiotics: 23.5% of early response, 29.4% of delayed response, and 47.1% of failure pattern. The percentage of failure was highest in mycobacterium and fungal infection, followed by* Escherichia coli* (ESBL), MRSA, mixed organism,* Pseudomonas *species, and* Enterobacter *species.

### 3.3. Dialysate WBC Change

**Figure 2:**
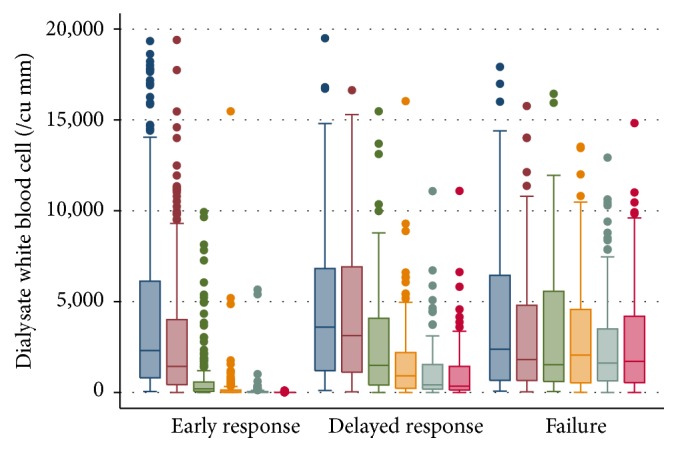
Boxplot of dialysate white blood cells in days 0–5 after antibiotic treatment, categorized by early response, delayed response, and failure groups.

**Figure 3:**
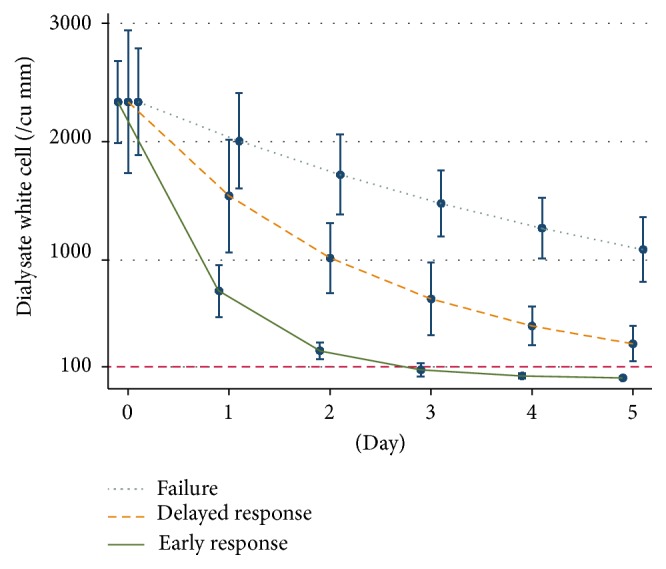
Pattern of dialysate white blood cell change, geometric mean of white blood cells, and standard error bar, categorized by early response, delayed response, and failure groups (parametric plot adjusted for baseline white blood cells). Red dashed line refers to the response level of dialysate white cell.

We presented the amounts of dialysate WBC five days after treatment in a boxplot graph due to the right skewed distribution of WBC (Figure 2). Log-transformed WBC was used to calculate a geometric mean and also standard error which were 2,202 ± 348 cells/mm^3^ in early response, 3,024 ± 603 cells/mm^3^ in delayed response, and 2,195 ± 452 cells/mm^3^ in failure group on the first day of treatment (Table 3). A constant trend in all groups was a reduction of dialysate WBC over time of treatment (Figure 3). We used multilevel linear regression, which had been adjusted for baseline white blood cells, to explore the average rate reduction per day in all three groups (early response, delayed response, and failure) which were 68.4% (95% CI, 67.4–69.3%), 34.0% (95% CI, 30.7–37.0%), and 14.2% (95% CI, 9.8–18.3%), respectively (*p* value < 0.001 for all comparisons) (Table 2). Internal validation using bootstrapping technique also revealed similar rate reduction in all groups (68.1% (95% CI, 67.5–68.7%) in early response, 30.8% (95% CI, 28.7–32.9%) in delayed response, and 12.9% (95% CI, 10.1–15.1%) in failure groups).

## 4. Discussion

This was the study exploring the change of dialysate WBC in order to represent the patterns of response to initial antibiotic treatment. We categorized episodes of peritonitis into 3 groups (early response, delayed response, and failure) and our data can confirm the definite patterns, which was derived from the mathematic model of log-transformed WBC counts over the treatment time. Each pattern showed the gradual decline of dialysate WBC, but with varying rates. By using these patterns, clinicians can predict whether each treatment episode will be success or failure.

Our patterns of WBC change are practical and match with the real clinical practice. We developed this model based on the first 5 days after antibiotic treatment because it represents the effectiveness of the empirical therapy and clinicians can use this information to guide further treatment. There were two previous reports exploring the pattern of dialysate WBC change. First, Dong et al. [12] categorized dialysate WBC patterns over the seven days of treatment into four groups based on a disease severity score [13], which was calculated by the sum of points for abdominal pain and fever at the time of diagnosis of dialysate-related peritonitis. One point increase in the disease severity score at baseline was correlated with higher number of WBC when compared between groups. Unfortunately, these patterns failed to predict either peritonitis-related death or transfer to hemodialysis. One year later, the same group presented additional data of WBC count patterns which categorized peritonitis episodes by the trend of WBC change in five days into four groups: group A (WBC count persistently declined), group B (WBC count declined after a transient increase), group C (WBC count increased after a transient decline), and group D (WBC count persistently increased). This report showed that WBC change does not always change in one direction. They found that the causative organisms were statistically different between groups. Gram-positive infection was found the most in group A but Gram-negative infection was rare. Groups A and B had a lower risk of treatment failure (11.8 and 16.4%, resp.); on the other hand groups C and D had higher failure rate (66.7 and 50%, resp.). The limitation of this report was a small sample size and excluded a large number of patients due to missing data that may not be able to extrapolate to other settings. Unlike the previous studies, our data categorized patterns by using a different approach because we did the analysis based on the outcomes of therapy and we use the mathematic model to calculate the average trend of WBC change without considering the fluctuation of WBC. Our study confirmed the association between the pattern of response and the type of causative microorganism. Gram-negative bacteria were found more in the delayed response group whereas mixed organisms, tuberculosis, and fungus appeared with a higher frequency in the failure group.

Gram-positive organism remains the major causative pathogen of peritoneal dialysis-related peritonitis in several studies [7, 9, 14, 15].* Staphylococcus epidermidis* was the most common causative organism but the virulence of this organism is low. On the contrary,* Staphylococcus aureus* either methicillin-sensitive or methicillin-resistant often caused severe episodes of peritonitis [16]. One study showed that MRSA increased the failure rate of peritoneal dialysis and also hospitalization rate by double when compared to other Gram-positive organisms [17]. Our study revealed the same findings. The onset of response in our study could represent the severity of each organism. We found that most episodes of* Staphylococcus epidermidis* and other* Staphylococcus (not Staphylococcus aureus)*,* Streptococcus viridan*, and* Streptococcus *group D (non-*Enterococcus)* had the early response pattern. Conversely, the delayed response and failure patterns were found more in* Staphylococcus aureus* and* Enterococcus* infection. These data were similar to the study of O'Shea et al. [18] and Edey et al. [19] data.

*Pseudomonas* species is a group of the Gram-negative bacteria that lead to severe peritoneal dialysis-related peritonitis. Szeto et al. [20] found that the primary response rate, which was defined as resolution of abdominal pain and dialysate neutrophil count of less than 100 cell/mm^3^ on day 10 with antibiotic, was 60.6% and complete cure rate, which was defined as complete cure with antibiotic alone within 120 days, was 22.1% in* Pseudomonas* peritonitis. There were 14 cases that had Tenckhoff catheter removed immediately when primary response could not be achieved and 10 of 20 cases with delayed Tenckhoff catheter removal after salvage antibiotics. Siva et al. [21] also found the rate of peritoneal catheter removal of 44% in* Pseudomonas* peritonitis, compared to 20% of non-*Pseudomonas* peritonitis. In the same way, our study showed that failure pattern was greater in Gram-negative infection especially in* Pseudomonas aeruginosa* and also in* Escherichia coli* (ESBL),* Acinetobacter baumannii*, and* Enterobacter* species infection.

From our point of view, the pattern of response will be helpful in 2 situations. First, when symptoms of patients relieve, but the dialysate WBC still persists more than 100 cells/mm^3^ on day 5 after antibiotic treatment and clinicians believe that those patients are in the delayed response group. If the rate of dialysate WBC reduction is about 34% (95% CI, 30.7–37), clinicians can wait and see instead of immediate removal of Tenckhoff catheter, whereas when the rate of dialysate WBC decline is less than 14.2%, it would suggest the failure of antibiotic treatment and a further management decision should be planned. Second, when the causative organism cannot be identified, the pattern of WBC change may help to predict the type of causative organism. We found that delayed pattern of response was associated with unique pathogens such as* Staphylococcus aureus*,* Pseudomonas aeruginosa*,* Enterobacter *species, and some drug-resistant organisms. Clinicians should combine these information with local prevalence of responsible organism and prescribe the appropriate salvage antibiotics. However, close follow-up is also important to help identify the patients who may have to stop peritoneal dialysis if the failure pattern occurs or another indication is present.

The strength of our report was large sample sizes and we classified the groups that matched with routine clinical practice. Furthermore, we used the multilevel mixed effect model to cope with serial measurement of dialysate WBC and repeated episodes of peritonitis in the same patients and we also had internal validation of these patterns which help make a more accurate conclusion. Nevertheless, there were some limitations. First, we excluded 83 medical records (11.4%) due to incomplete data. But we thought our sample was adequate to explore the patterns of WBC with less selection bias. Second, the percent reduction of WBC in each group could only be interpreted as an average trend of change. We suggested using the pattern with clinical correlation. Third, this study focused on the pattern of dialysate WBC in aspect of short term outcomes. Long-term follow-up in each pattern would be done and reported in further studies. Finally, the retrospective nature of this study needs to be considered when interpreting these results. We believed that this study will generate the idea of how important WBC patterns are. Prospective study should be done to confirm our hypothesis.

## 5. Conclusion

In conclusion, our study determined the patterns of WBC response after empirical antibiotic therapy in peritoneal dialysis-related peritonitis patients which could be categorized into early response, delayed response, and failure groups. These findings may guide clinicians to decide a proper treatment whether to continue medical therapies or to aggressively remove the peritoneal catheter.

## Figures and Tables

**Table 1:** Clinical characteristics of peritoneal episodes, categorized by early response, delayed response, and failure groups (455 patients; 644 episodes).

Characteristics	Early response (*N* = 378 )	Delayed response (*N* = 122)	Failure (*N* = 144)	*p* value
Male, *n* (%)	192 (50.8)	58 (47.5)	70 (48.6)	0.789
Age, year	62.1 ± 12.0	60.2 ± 13.5	59.3 ± 13.1	0.051
Diabetes, *n* (%)	246 (65.1)	79 (64.8)	97 (67.4)	0.871
HIV, *n* (%)	4 (1.1)	0	3 (2.1)	0.275
Primary kidney disease, *n* (%)				
Diabetic nephropathy	245 (64.8)	78 (63.9)	96 (66.7)	0.890
Glomerulonephritis	3 (0.8)	1 (0.8)	2 (1.4)	
Nephrosclerosis	60 (15.9)	15 (12.3)	21 (14.6)	
Obstructive uropathy	3 (0.8)	0	1 (0.7)	
Others	18 (4.8)	9 (7.4)	9 (6.3)	
Unknown	49 (13.0)	19 (15.6)	15 (12.9)	
Dialysis vintage, month				
Median [min–max]	16.8 [0–87.6]	15.0 [0–76.8]	19.8 [0–81.6]	0.309
First episode of peritonitis	218 (57.7)	64 (52.5)	71 (49.3)	0.190
Episode of peritonitis				
Median [min–max]	1 [1–8]	1 [1–8]	2 [1–9]	0.494
Body temperature, Celsius	37.1 ± 0.9	37.0 ± 0.9	37.0 ± 1.0	0.520
Empirical antibiotic regimen				
Cefazolin	333 (88.1)	111 (91.0)	127 (88.2)	0.701
Vancomycin	17 (4.5)	5 (4.1)	8 (5.6)	0.869
Ceftazidime	335 (88.6)	113 (92.6)	131 (91.0)	0.423
Gentamicin/amikacin	12 (3.2)	1 (0.8)	1 (0.7)	0.167
Meropenem	6 (1.6)	2 (1.6)	6 (4.2)	0.222
Cefepime	23 (6.1)	5 (4.1)	4 (2.8)	0.284
Organism				
Culture negative	106 (28.0)	39 (32.0)	46 (31.9)	<0.001
Gram-positive	161 (42.6)	33 (27.1)	19 (13.2)	
Gram-negative	92 (24.3)	45 (36.9)	40 (27.8)	
Mixed organism	19 (5.0)	4 (3.3)	14 (9.7)	
Tuberculosis	0	0	9 (6.3)	
Fungus	0	1 (0.8)	16 (11.1)	
Empirical antibiotic success^*∗*^				
In total cases	375 (99.2)	36 (29.5)	0	<0.001
In culture negative cases	106/106 (100)	19/39 (48.7)	0	<0.001

^*∗*^Empirical antibiotic success; success in treatment without changing to any salvage antibiotic regimens.

**Table 2:** Predictive value of early response, delayed response, and failure groups according to types of organism.

Type of organism	Number of episodes	Early response (*N* = 378)	Delayed response (*N* = 122)	Failure (*N* = 144)
Culture negative	191	106 (55.5)	39 (20.4)	46 (24.1)
Gram-positive organism	213	161 (75.6)	33 (15.5)	19 (8.9)
* Staphylococcus epidermidis *	70	52 (74.3)	10 (14.3)	8 (11.4)
* Staphylococcus aureus *	21	8 (38.1)	9 (42.9)	4 (19.1)
* MRSA *	7	1 (14.3)	3 (42.9)	3 (42.9)
Other* Staphylococcus *	5	5 (100)	0	0
* Streptococcus viridan *	21	20 (95.2)	1 (4.8)	0
* Streptococcus *group D	42	38 (90.5)	4 (9.5)	0
* Enterococcus *	9	6 (66.7)	2 (22.2)	1 (11.1)
Other *Streptococcus*	35	29 (82.9)	3 (8.6)	3 (8.6)
Other Gram-positive organisms	3	2 (66.7)	1 (33.3)	0
Gram-negative organism	177	92 (52.0)	45 (25.4)	40 (22.6)
* Escherichia coli *	57	34 (59.7)	15 (26.3)	8 (14.0)
* Escherichia coli *(ESBL)	17	4 (23.5)	5 (29.4)	8 (47.1)
* Klebsiella *species	40	23 (57.5)	11 (27.5)	6 (15.0)
* Pseudomonas aeruginosa/*spp.	15	7 (46.7)	3 (20.0)	5 (33.3)
* Acinetobacter baumannii/*spp.	18	8 (44.4)	5 (27.8)	5 (27.8)
* Enterobacter *spp.	15	6 (40.0)	4 (26.7)	5 (33.3)
Other Gram-negative organisms	15	10 (66.7)	2 (13.3)	3 (20.0)
Mixed organism	37	19 (51.2)	4 (10.8)	14 (37.8)
Tuberculosis	9	0	0	9 (100)
Fungus	17	0	1 (5.9)	16 (94.1)

**Table 3:** Dialysate white blood cell count five days after antibiotics treatment, categorized by early response, delayed response, and failure groups.

Day of treatment	Dialysate WBC, cell/mm^3^ (geometric mean ± SE)		
	Early response (*N* = 378)	Delayed response (*N* = 122)	Failure (*N* = 144)
Day 0	2202 ± 348	3024 ± 603	2195 ± 452
Day 1	1174 ± 220	2650 ± 476	1633 ± 403
Day 2	164 ± 72	1028 ± 307	1494 ± 340
Day 3	27 ± 54	613 ± 295	1264 ± 278
Day 4	9 ± 25	409 ± 164	1004 ± 255
Day 5	3 ± 1	345 ± 151	895 ± 274
% reduction (developed model)^*∗*^	68.4 [67.4, 69.3]	34.0 [30.7, 37.0]	14.2 [9.8, 18.2]
% reduction (bootstrapping model)^*∗*^	68.1 [67.5, 68.7]	30.8 [28.7, 32.9]	12.9 [10.1, 15.1]

WBC: white blood cells; % reduction presented in average rate per day [95% confident interval].

^*∗*^ *p* value < 0.001 for all comparisons by multiple comparison under multilevel modeling.
